# Health care trajectories and medication consumption of substance users in treatment: linking TDI and IMA databases (Belgium)

**DOI:** 10.1186/2049-3258-73-S1-P30

**Published:** 2015-09-17

**Authors:** Karin De Ridder, Jérôme Antoine, Lies Gremeaux, Els Plettinckx, Peter Blanckaert, Jean Tafforeau

**Affiliations:** 1 Scientific Institute of Public Health (WIV-ISP), Brussels, Belgium

## Background

As problem substance use has a relative low prevalence and is often socially stigmatised, it is rather difficult to study health care trajectories and medication consumption among substance users. In Belgium, the Treatment Demand Indicator register (TDI) is a major information source for drug epidemiology. However, it should be considered as an incidence register as only new drug-related treatment episodes are registered. Linkage with national compulsory health insurance data (IMA) will create a data source in which longitudinal and case-control studies can be performed.

## Methods

In the TDI register, every new treatment episode started by a person in a treatment centre for his/her alcohol or illicit drug use is registered. This research project will comprise TDI data (2011-2014) with the client's sociodemographic profile, substance use pattern and treatment information linked to a 10-years period of IMA data (2008-2017). IMA collects the data from the seven Health Insurance Organizations in three databases: (1) population database, (2) reimbursed health care, (3) reimbursed prescribed medicines. A Trusted Third Party (eHealth) is to link the coded version of the unique national identification number (NIN) of both registers. Each TDI case will be matched by sex, age and municipality with four controls who have no administrative registrations of drug treatment in the TDI and IMA registers. The research project has been approved by the Privacy Commission (SCSZG/15/033) and the linkage will be executed from autumn 2015.

## Results

The TDI register (2011-2014) contains 50,291 persons and 69% (N=34,628) clients with a unique NIN are eligible for linkage. Among them, 75% were male. In 41% of the registered cases, the primary substance was alcohol, followed by cannabis (21%), opiates (17%), cocaine (9%) and stimulants other than cocaine (7%).

## Conclusion

A linkage of TDI-IMA will result in the first large-scaled longitudinal database in the Belgian drug epidemiology.

